# A Photochromic Azobenzene Peptidomimetic of a β-Turn Model Peptide Structure as a Conformational Switch

**DOI:** 10.3389/fchem.2019.00180

**Published:** 2019-03-29

**Authors:** Francesca Nuti, Cristina Gellini, Maud Larregola, Lorenzo Squillantini, Riccardo Chelli, Pier Remigio Salvi, Olivier Lequin, Giangaetano Pietraperzia, Anna Maria Papini

**Affiliations:** ^1^Laboratory of Peptide and Protein Chemistry and Biology (PeptLab), Sesto Fiorentino, Italy; ^2^Department of Chemistry “Ugo Schiff”, University of Florence, Sesto Fiorentino, Italy; ^3^PeptLab@UCP Platform and Laboratory of Chemical Biology EA4505, Université Paris-Seine, Cergy-Pontoise, France; ^4^European Laboratory for Non-Linear Spectroscopy (LENS), Sesto Fiorentino, Italy; ^5^Laboratory of Biomolecules, CNRS, Sorbonne University, Ecole Normale Superieure, PSL University, Paris, France

**Keywords:** azobenzene, cis/trans photoisomerization, photoswitchable peptide, optical control, NMR spectroscopy, UV/Vis spectroscopy

## Abstract

The insertion of azobenzene moiety in complex molecular protein or peptide systems can lead to molecular switches to be used to determine kinetics of folding/unfolding properties of secondary structures, such as α-helix, β-turn, or β-hairpin. In fact, in azobenzene, absorption of light induces a reversible trans ↔ cis isomerization, which in turns generates a strain or a structure relaxation in the chain that causes peptide folding/unfolding. In particular azobenzene may permit reversible conformational control of hairpin formation. In the present work a synthetic photochromic azobenzene amino acid derivative was incorporated as a turn element to modify the synthetic peptide [Pro^7^,Asn^8^,Thr^10^]CSF114 previously designed to fold as a type I β-turn structure in biomimetic HFA/water solution. In particular, the P-N-H fragment at positions 7–9, involved in a β-hairpin, was replaced by an azobenzene amino acid derivative (synthesized *ad hoc*) to investigate if the electronic properties of the novel peptidomimetic analog could induce variations in the isomerization process. The absorption spectra of the azopeptidomimetic analog of the type I β-turn structure and of the azobenzene amino acid as control were measured as a function of the irradiation time exciting into the respective first ππ^*^ and nπ^*^ transition bands. Isomerization of the azopeptidomimetic results strongly favored by exciting into the ππ^*^ transition. Moreover, conformational changes induced by the *cis*↔ *trans* azopeptidomimetic switch were investigated by NMR in different solvents.

## Introduction

Azobenzene has been recognized as a potential molecular photoswitch in various fields, such as polymer science, material science, chemistry, and life sciences (Marchi et al., 2012; Goulet-Hanssens and Barrett, 2013; Dong et al., 2015; Bushuyev et al., 2018; Miniewicz et al., 2018). Azobenzene amino acids protected for solid phase synthesis proposed by Sewald et al. opened possibility the for incorporation into photoswitchable peptides (Juodaityte and Sewald, 2004; Renner and Moroder, 2006; Aemissegger and Hilvert, 2007). In particular, compounds containing a methyl spacer between the phenyl ring and the amino group, as [3-(3-aminomethyl)-phenylazo]phenylacetic acid (AMPP) acid or (4-aminomethyl)phenylazobenzoic acid (AMPB), give more flexibility to the chemical structure of a Xaa–AMPB–Yaa fragment than the rigid chromophore, 4-[(4-amino)phenylazo]benzoic acid (APB).

When inserted into larger molecular systems, like protein chains or peptides, azobenzene can be used as a molecular switch and/or as a probe to determine kinetics of folding/unfolding of the corresponding secondary structure, like α-helix, β-turn, or β-hairpin (Behrendt et al., 1999; Kumita et al., 2000; Renner et al., 2000; Spörlein et al., 2002; Komarov et al., 2018). In fact, absorption of light by azobenzene induces a reversible *trans* ↔ *cis* isomerization, which in turns generates a strain or a structure relaxation in the chain that causes peptide unfolding/folding. The process can be easily monitored looking at the absorption spectrum of azobenzene itself, that is strongly different in the two forms, *trans* and *cis* (Schultz et al., 2003; Satzger et al., 2004; Quick et al., 2014). In particular, the AMPP chromophore incorporation in a peptide sequence led to a β-hairpin structure after irradiation (Dong et al., 2006, 2017; Rampp et al., 2018), and the AMPB was described as a trigger molecule in cyclic peptide structures (Ulysse et al., 1995; Renner et al., 2005).

When a chromophore unit is integrated into a linear or cyclic peptide, the *trans* ↔ *cis* isomerization of an azobenzene derivative induced by UV/VIS photo-irradiation, has been demonstrated to induce a reversible change in the peptide structure modulating its biological activity (Ali et al., 2015). For example azobenzene amino acid has been reported for antigen-antibody photocontrol (Beharry et al., 2008; Parisot et al., 2009). In a cyclic polypeptide ligand, azobenzene *trans* ↔ *cis* isomerization induced drastic changes in recognition by neural NO synthase leading to reversible photocontrol of muscle fibers (Hoppmann et al., 2011). Finally, in an amyloid azobenzene containing peptide, isomerization plays an active role in self-assembly into β-amyloid fibrils (Deeg et al., 2011).

β-Hairpins are very interesting structures as they are involved in many biological processes i.e., often constitute binding epitopes and are implied in protein–protein or protein–DNA interactions (Hillier et al., 1999; Gajiwala et al., 2000; Wong et al., 2000; Schumacher et al., 2001; Zavala-Ruiz et al., 2004). Thus, the development of highly stable β-hairpins based on introduction of molecules as azobenzene allowed to control the hairpin structure and initiate a folding or unfolding transition with high isomerization yield, remarkable photostability, and ultra-fast kinetics.

In previous studies, the family of structure-based designed β-hairpin peptides termed CSF114(Glc) has been developed to expose aberrant post-translational modifications (PTMs) to characterize antibodies as biomarkers of autoimmune diseases in patient sera (Lolli et al., 2005a,b; Papini, 2009; Pandey et al., 2012). In fact, we demonstrated that the β-turn structure is crucial for the correct exposure of the PTM and allows a specific and high affinity antibody interaction in the context of solid-phase immunoenzymatic assays (Carotenuto et al., 2006, 2008). In this work, a modified sequence of [Pro^7^,Asn^8^,Thr^10^]CSF114 was selected as an optimized type I β-turn structure. Aim of the present work is the design and synthesis of a photocontrolled probe, based on AMPB azobenzene as a turn element in the central part of the amino acid sequence, to investigate if the electronic properties of the new molecule could induce variations in the isomerization process of the azobenzene unit and to study the effect of the photoswitch on its conformation.

## Materials and Methods

### Reagents

All Fmoc-protected amino acids, Fmoc-Wang resins, DIC (*N,N*′-Diisopropylcarbodiimide), and Oxyma were purchased from Iris Biotech GmbH (Marktredwitz, Germany). The following amino acid side-chain-protecting groups were used: OtBu (Asp, Glu), tBu (Ser, Thr), Pbf (Arg), Trt (Gln, His), and Boc (Lys). Peptide-synthesis grade *N*,*N*-dimethylformamide (DMF) was purchased from Scharlau (Barcelona, Spain); acetonitrile (ACN) from Carlo Erba (Milan, Italy); dichloromethane (DCM), trifluoroacetic acid (TFA), piperidine were purchased from Sigma-Aldrich (Milan, Italy).

### MW-Assisted Solid-Phase Peptide Synthesis

The azopeptide **1** was synthesized by microwave-assisted solid-phase peptide synthesis (MW-SPPS) following the Fmoc/tBu strategy, using the Liberty Blue™ automated microwave peptide synthesizer (CEM Corporation, Matthews, NC, USA) following the protocol described elsewhere (Rizzolo et al., 2011). The resin used was a Fmoc-Lys(Boc)-Wang (loading 0.24 mmol/g). Modified amino acids were introduced using the synthesized protected building-blocks suitable for Fmoc/tBu SPPS (Paolini et al., 2007; Rentier et al., 2015). Coupling was performed with the azobenzene amino acid AMPB (2.5 eq), HATU as activator (2.5 eq), and DIPEA (3.5 eq) for 30 min at room temperature. Uncertain peptide coupling steps were checked by the ninhydrin test as described by Kaiser (Kaiser et al., 1970), or micro-cleavages performed with a microwave apparatus CEM Discover™ single-mode MW reactor (CEM Corporation, Matthews, NC, USA). Final cleavage was performed using a mixture of TFA/TIS/H_2_O (95:2.5:2.5 v:v:v) for 3 h at room temperature.

The crude azopeptide was pre-purified by Reverse Phase Liquid Chromatography (RP-HPLC) using a Li-Chroprep C-18 column on an Armen Instrument (Armen Instrument, Saint-Avé, France) working at 20 ml/min with H_2_O (MilliQ) and CH_3_CN as solvent systems. The second step of purification was performed by semipreparative RP-HPLC on a Waters instrument (Separation Module 2,695, detector diode array 2,996) using a Phenomenex (Torrance, CA, USA) Jupiter column C18 (10 μm, 250 × 10 mm), at 4 mL/min with solvent systems A (0.1% TFA in H_2_O) and B (0.1% TFA in CH_3_CN).

The azopeptide **1** was characterized by RP-HPLC-ESI-MS, obtaining a final purity ≥ 98% (Figure S1). HPLC: t_r_ = 3.17 min (*cis* isomer) and 3.78 min (*trans* isomer), gradient 35–55% of B in 5 min; Mr = calcd. for C_116_ H_170_ N_29_ O_25_ S_1_: 2402,88 ESI-MS: m/z: 1202,47 [M+2H]^2+^; 802,02 [M+3H]^3+^ RP-HPLC system is an Alliance Chromatography (Waters, Milford Massachusetts, USA) with a Bioshell A160 C18 (Sigma Aldrich, Milano Italy; 1.7 μm 2.1 × 50 mm) column at 35°C, at 0.6 mL/min coupled to a single quadrupole ESI-MS Micromass ZQ (Waters, Milford Massachusetts, USA). The solvent systems used were A (0.1% TFA in H_2_O) and B (0.1% TFA in CH_3_CN). Peptides were lyophilized using an Edward Modulyo lyophilizer (Richmond scientific Ldt., Lancashire, Great Britain).

### UV/Vis Spectra Experiments

The experimental set up used for the irradiation procedure consists of a Xe “Ozone free” Orion lamp emitting 450 W in the spectral range 200–2,000 nm. The light is focalized through a lens (f = 200 mm) onto the entrance slit of a monochromator. The exit beam, having 1 nm bandwith, is shaped by means of a pin-hole and collimated with a 50 mm lens on the sample cuvette (quartz, 1 cm optical path length). A magnetic stirrer placed inside the cuvette ensures that the irradiating beam always interacts with fresh solution. Incident beam cross section has been estimated 0.8 × 0.8 cm^2^. Incident power has been measured with a Coherent Field Max II power meter. The power we used for the irradiation was around 500 μW for all the three excitation wavelengths used 313, 440, and 330 nm. Absorption spectra have been obtained with a Varian Cary 5 spectrophotometer, with 2 nm bandwith resolution.

Azobenzene was purchased by Sigma-Aldrich (purity 98%). [(4-aminomethylphenyl)diazenyl] phenylacetic acid was synthesized as described by Juodaityte and Sewald (2004) and azopeptide **1** was synthesized as described. Solution concentrations were all around 10^−4^ M, using ethanol (Merck, purity grade: Uvasol) as a solvent. Unknown molar extinctions were determined for the new synthetic azopeptide **1** in the *trans* form (amino derivative ε_t,(326)_ = 9,000 cm^−1^M^−1^; aminopeptide ε_t,(330)_ = 5,090 cm^−1^M^−1^). The extinctions of the corresponding *cis* form was calculated considering still valid the ratio ε_cis_/ε_trans_ in azobenzene **3**.

### NMR Experiments

NMR experiments were recorded on a 500 MHz Bruker Avance III spectrometer (Wissembourg, France) equipped with a TCI ^1^H/^13^C/^15^N cryoprobe. The lyophilized azopeptide **1** was dissolved in 550 μL of 50% TFE-d_3_/50% H_2_O, 50% TFE-d_3_/50% D_2_O, or 50% ACN-d_3_/50% H_2_O at ~0.5 mM concentration for experiments before irradiation. For induction of azobenzene *trans* ↔ *cis* isomerization, the sample was irradiated in 100% TFE-d_3_ or 50% ACN-d_3_/50% H_2_O with a VL-6.L UV-lamp (Vilber, Germany) emitting 6 W at 365 nm for 1–5 h. Suitable volumes of solvents were added after irradiation to recover the same solvent conditions as for experiments before irradiation. ^1^H and ^13^C resonance assignments were obtained from the analysis of 2D ^1^H-^1^H TOCSY (DIPSI2 isotropic scheme of 66 ms duration), ^1^H-^1^H NOESY (200 or 400 ms mixing time), ^1^H-^1^H ROESY (250, 300, or 400 ms mixing time), ^1^H-^13^C HSQC, and ^1^H-^13^C HSQC-TOCSY. NMR experiments were processed with TOPSPIN 3.5 (Bruker) and analyzed with NMRFAM-Sparky program (Lee et al., 2015). NMR chemical shifts were calibrated with respect to the residual protiated solvent signal on 1D ^1^H or 2D ^1^H-^13^C HSQC experiments.

## Results and Discussion

In this work we designed, synthesized and studied the reversible *cis* ↔ *trans* photoisomerization of the [Pro^7^,Asn^8^,Thr^10^]CSF114 analog peptide **1**, where from the original sequence the P-N-H tripeptide was replaced by the photoswitch (4-aminomethyl)phenylazobenzoic acid (AMPB). The photoisomerization of the synthetic azopeptide **1** was explored and its reversibility was compared with more simple systems, the azobenzene amino acid **2** and the azobenzene **3**.

**Design of the azopeptide 1**. In the literature photochromic compounds, i.e., azobenzene, have been reported as molecules able to isomerize reversibly, when exposed to light of appropriate wavelength.

The 21-mer peptide [Pro^7^,Asn^8^,Thr^10^]CSF114, derived from the family of structure-based designed β-turn peptides termed CSF114(Glc), is characterized by a type I β-turn motif (Carotenuto et al., 2006, 2008). In this sequence the β-turn structure was shown around the proline and asparagine residues in positions 7-8. The role of conformation in the recognition and binding of this synthetic antigenic probe to autoantibodies in the context of an immunoenzymatic assay (ELISA) was previously determined to be fundamental.

**Figure 1:**
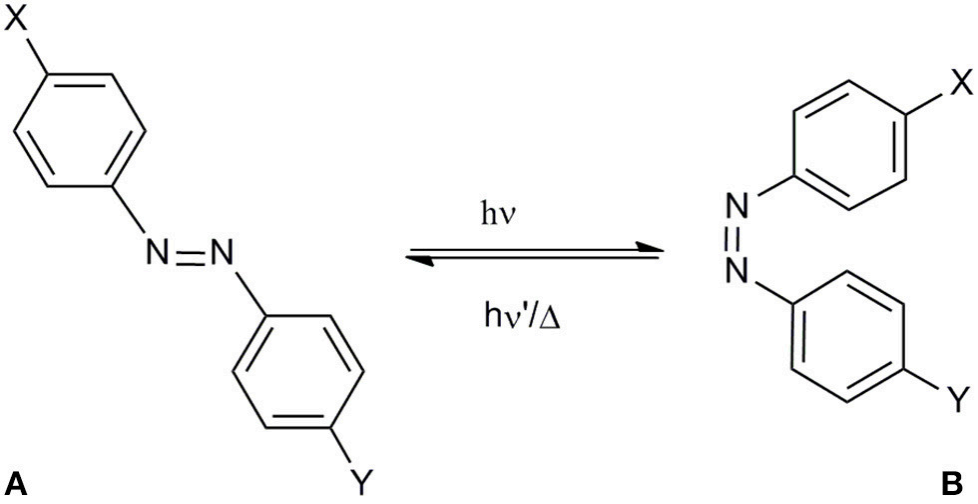
**(A)** azopeptide **1** (X = T-P-R-V-E-R-;Y = T-V-F-L-A-P-Y-G-W-M-V-K); *trans*-(4-aminomethyl)phenylazobenzoic (AMPB) acid (X = CH_2_NH_2_,Y = CH_2_COOH) **2**; *trans-*azobenzene (X,Y = H) **3**, **(B)** their possible *cis* conformation.

Because of the importance of the conformation and of the correct exposure of epitopes involved in autoantibody recognition, the light-induced conformational change of the synthetic peptide [Pro^7^,Asn^8^,Thr^10^]CSF114, after the introduction of the azobenzene moiety into the sequence is the object of the present study (Figure 1). Starting from the [Pro^7^,Asn^8^,Thr^10^]CSF114 sequence, the P-N-H segment was targeted for replacement by an AMPB azobenzene amino acid, as a turn element.

The photoswitch (4-aminomethyl)phenylazobenzoic acid (AMPB) was obtained by the condensation of a 4-nitrophenylacetic acid with 4-aminobenzylamine as described previously (Ulysse and Chmielewski, 1994; Juodaityte and Sewald, 2004; Aemissegger et al., 2005). The amino function of 4-aminobenzylamine was protected as Fmoc to obtain 4-[2-[4-[[[(9H-fluorenyl-9-methoxy)carbonyl]amino]methyl]phenyl]diazenyl]benzenacetic acid to be used in Fmoc solid-phase peptide synthesis. Its incorporation into the peptide sequence proceeded into a straightforward manner applying the standard Fmoc-solid phase methodology, using HATU as coupling reagent. The synthesis of the azobenzene-containing peptide **1** was carried out on a 0.1 mmol scale following the standard Fmoc/tBu solid phase peptide synthesis (SPPS) starting from Fmoc-Lys(Boc)-Wang resin. After coupling the amino acids of the sequence protected for SPPS, the peptide was cleaved from the resin and purified and characterized by RP-HPLC coupled to Mass Spectrometry. The ability of AMPB to induce variations in the isomerization process was elucidated by UV-Vis and NMR.

### The Azobenzene Peptide as Molecular Switch

**Figure 2:**
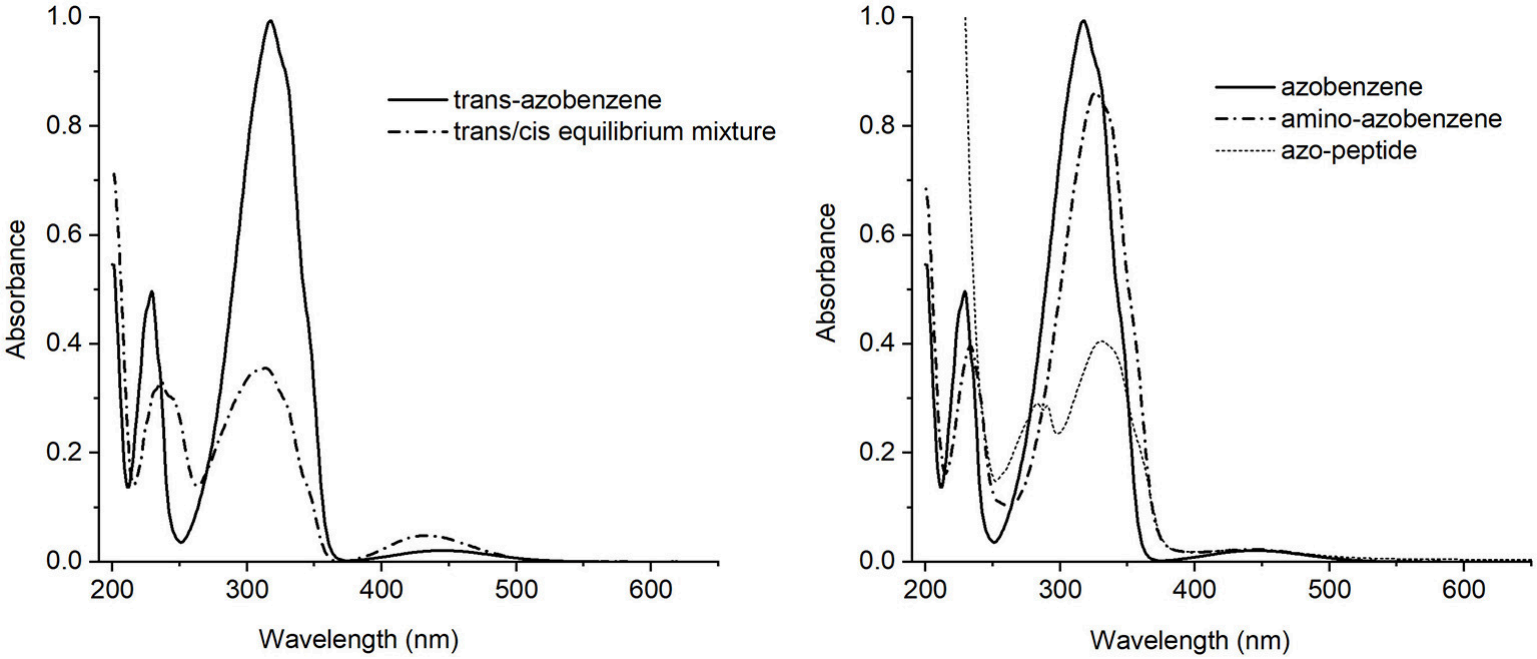
**Left side:** UV-Vis absorption spectra of the *trans*-azobenzene **3** (solid line) and the equilibrium mixture *trans*/*cis* (dash-dotted line). **Right side:** UV-Vis absorption spectra of the *trans* isomers of azobenzene **3** (solid line), aminoazobenzene **2** (dash-dotted line) and azopeptide **1** (short dash line).

In order to propose the synthetic azobenzene as a molecular switch, a deep characterization of its response to light excitation is mandatory to fully understand the effect of substituents on isomerization (Figure 2).

The *trans*-azobenzene **3** spectrum showed in Figure 2 (left), is characterized by a strong absorption centered around 317 nm and a medium one at 230 nm, both assigned as ππ^*^ electronic transitions. At longer wavelength, about 440 nm, a weak band is observed due to the nπ^*^ state. Appearance of the *cis* form is revealed by the intensity decrease of the main band and a simultaneous growing of a medium intensity absorption at 238 nm.

The absorption spectra of the *trans* isomers of amino-azobenzene **2** and azopeptide **1** are shown in Figure 2 (right). While the correspondence of the electronic transitions is maintained, a small red shift of the bands is observed in the amino-derivative probably due to a moderate conjugation and/or electron-donor effect induced by the substituents on the aromatic rings. In the azopeptide **1**, on the blue side of the aromatic ππ^*^ transition whose maximum is red-shifted at 330 nm, the characteristic absorption of tryptophan is also observed at 290 nm.

**Figure 3:**
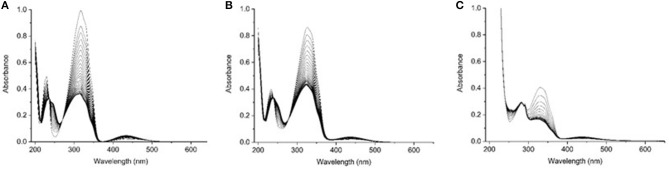
UV-Vis absorption spectra at several irradiation time for **(A)** azobenzene **3**, **(B)** aminoazobenzene **2**, and **(C)** azopeptide **1**, exciting at the wavelength of the absorption maxima (313 nm for azobenzene and aminoazobenzene, and 330 nm for the azopeptide **1**).

Addition of substituents to azobenzene affects the electronic distribution and can also modify the *trans-cis* isomerization process and its photochemical yields (Crecca and Roitberg, 2006; Bandara and Burdette, 2012). Therefore, the absorption spectra of the three compounds have been measured as a function of the irradiation time, exciting into the respective first ππ^*^ and nπ^*^ transition bands. For all the three examined compounds, the *trans*→*cis* conversion is more effective when excitation is performed into the maximum of the ππ^*^ band rather than into the nπ^*^. For this reason we focused our attention only on the *trans*→*cis* conversion obtained exciting into the ππ^*^ absorption band (Figure S2). The resulting absorption spectra of azobenzene, aminoazobenzene and azopeptide are shown in Figures 3A–C, respectively.

In the absorption spectra, isosbestic points are observed, indicating the presence of the *trans*-*cis* equilibrium in solution. Azobenzene absorption spectrum shows up to four isosbestic points, at 373, 270, 236, and 213 nm. On the opposite the azopeptide **1** shows only one isosbestic point at longer wavelength, due to the overlap in the blue side of the spectrum, with the large tryptophan absorption. In all three cases, after some time, a photostationary state is obtained, where the *trans*→*cis* and *cis*→*trans* isomerization rates equalize and no further variation is observed in the absorption intensity.

**Figure 4:**
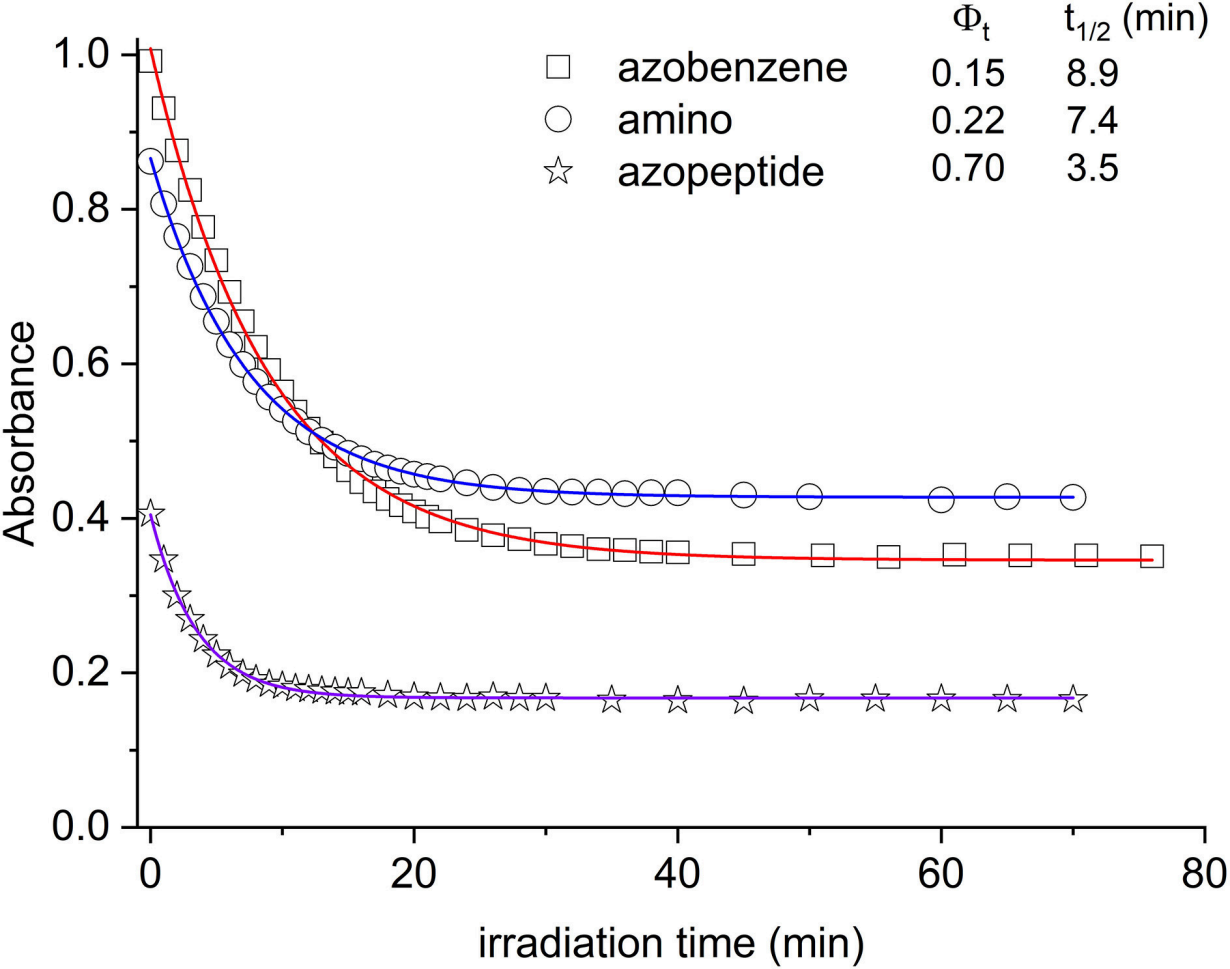
Absorbance decrease of the ππ* transition as a function of the irradiation time (azobenzene, squares; aminoazobenzene, circles; azopeptide **1**, stars). The time evolution is due to the conversion between the *trans* and *cis* forms. In the inset the photoconversion quantum yields are reported, along with the respective decay times obtained by fitting the absorbance data (solid lines in the graph).

In Figure 4, the absorption maxima are plotted as a function of the irradiation time at those wavelengths. From this graph, quantitative information can be gained about the isomerization kinetics and the relative photochemical yields, following known kinetic procedure (Zimmermann et al., 1958; Bortolus and Monti, 1979).

In Figure 4, the absorption maxima are plotted as a function of the irradiation time at those wavelengths. From this graph, quantitative information can be gained about the isomerization kinetics and the relative photochemical yields, following known kinetic procedure (Zimmermann et al., 1958; Bortolus and Monti, 1979). In this respect, we have considered the kinetic equation:

(1) $$\begin{array}{r} \frac{d\left[cis\right]}{dt}=\Phi_{t}I_{\lambda ,t}-\Phi_{c}I_{\lambda ,c}-k\left[cis\right] \end{array}$$

where [*cis*] = C_0_·Y, being C_0_ the initial molar concentration of the trans isomer and Y the *cis* molar fraction appearing in time. Φ_t_ is the photochemical yield of the *trans*→*cis* reaction, while Φ_c_ of the *cis*→*trans* one. *k* is the constant relative to the thermic isomerization. Since this last mechanism is much slower than the photochemistry, it can be neglected in our experiment (Zimmermann et al., 1958). I_λ_ is the power density absorbed by the *trans/cis* isomer. After some manipulations, the kinetic equation assumes the following aspect:

(2) $$\begin{array}{r} \frac{d\left[Y\right]}{dt}=\Phi_{t}I_{0,\lambda }\frac{\left[\varepsilon_{t}\left(Y_{\infty }-Y\right)\right]}{FY_{\infty }} \end{array}$$

where ε_t_ is the extinction coefficient of the *trans* form, *F* = *A/(1–10*^−*A*^*)*, being *A* the absorbance plotted in Figure 4. This last equation may be integrated giving a function whose time dependence is linear. The slope *m* of this line is related to the photochemical yield of the *trans* isomer (Zimmermann et al., 1958):

(3) $$\begin{array}{r} \Phi_{t}=\frac{-mY_{\infty }}{I_{0,\lambda }\varepsilon_{t}} \end{array}$$

By using this kinetic analysis, the photochemical yield Φ_t_ shown in the inset of Figure 4 is obtained.

In agreement with previous data (Bortolus and Monti, 1979; Siampiringue et al., 1987; Satzger et al., 2004), azobenzene photochemical yield in ethanol was found to be 0.15. In the amino derivative **2**, the ring substitution has the effect to increase the yield to 0.22. The major change is observed in the case of azopeptide 1, where the isomerization results strongly favored giving a yield of 0.70.

Adding electron-donor or electron-attractor groups to the aromatic rings alters the isomerization process due to the modification of the electron density on the molecule (Crecca and Roitberg, 2006; Bandara and Burdette, 2012). In the amino derivative AMBP, the presence of CH_2_ spacers reduces the electron-donor effect of the amino groups. Nevertheless, a small change in the isomerization yield is observed. Also in the azopeptide **1**, the ring substituents are bonded through CH_2_ spacers. However a strong increase in the *trans*–*cis* quantum yield value is observed according to a faster isomerization kinetics (Yamamura et al., 2014). Such a different behavior can then be ascribed to some effective interactions involving the amino acid residues of the side chains, which stabilize the *cis* form of the azobenzene moiety. However, from the absorption data, the nature of this interaction cannot be known. The NMR data suggest the presence of interactions between the azobenzene aromatic protons and the side chains of amino acid residues, influencing the isomerization kinetic and the thermodynamic parameters (Renner et al., 2000).

Further investigations on thermal *cis*→*trans* reconversion kinetics confirm the stability of the final compound, accordingly to the NMR results. In fact, keeping irradiated solutions of the azopeptide **1** 48 h in the dark at 50°C, only half *trans* form is thermally recovered, while for azobenzene and α-helix short peptide chain derivatives the recovery is complete, on this time scale, also at room temperature (Kumita et al., 2000).

### Conformational Analysis

**Figure 5:**
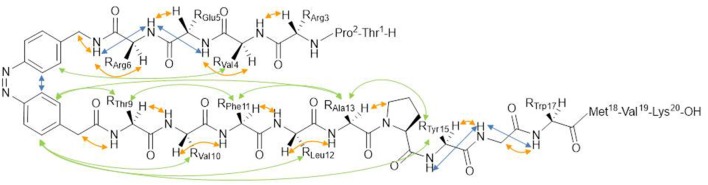
Structure of the *cis*-azopeptide **1** with observed NOEs indicated by arrows.

The effects of azobenzene *trans* ↔ *cis* isomerization on azopeptide **1** (Figure 5) conformation were investigated by NMR under different solvent conditions. Azopeptide **1** was found to be poorly soluble in water and organic solvents such as methanol and acetonitrile. Azopeptide was soluble in DMSO but exhibited poor spectrum quality with large H^N^ signals. A previous NMR study of CSF114 analogs exhibiting β-hairpin propensity was carried out in HFA:water mixture (1:1) to stabilize folded conformations (Carotenuto et al., 2008). However, in the case of azopeptide **1**, this solvent condition led to broad NMR signals that precluded conformational studies. We then turned to trifluoroethanol (TFE) as a different fluorinated cosolvent, which is commonly used to stabilize peptide structures. TFE/water 1:1 mixture yielded satisfactory quality of NMR spectra (Figures S3, S4). The stabilization of secondary structures by TFE is well-known for α-helices (Roccatano et al., 2002) but far less documented for β-hairpin structures (Blanco et al., 1994). Therefore, we also decided to investigate the use of ACN/water 1:1 mixture to analyze the effect of organic cosolvent on azopeptide folding.

Complete ^1^H assignments of the *trans* form were obtained both in TFE/water 1:1 and ACN/water 1:1 mixtures, using 2D TOCSY and 2D NOESY (or ROESY) experiments (Tables S1, S3, supporting information). Upon irradiation, the resonances of the *cis* form was observed on NMR spectra, with a population reaching 75–80% after 5 h irradiation. The *cis*→*trans* conversion was slow enough (>2 weeks) to record NMR spectra over several days, enabling to fully assign the *cis* form (Tables S2, S4). The *cis* form was characterized by a large (~1 ppm) upfield shift of aromatic *meta* resonances (with respect to methylene group) of azobenzene moiety, confirming the *trans*→*cis* isomerization of the N = N azo-bond (Figure S4).

**Figure 6:**
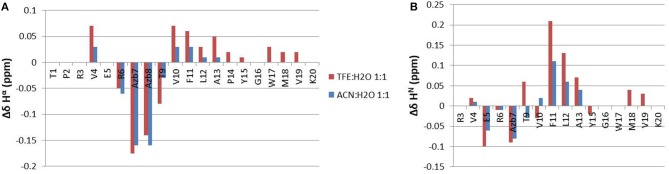
Δδ chemical shift differences between the *cis* and the *trans* forms of azopeptide **1**, calculated for H^α^ **(A)** and H^N^ protons **(B)**.

A comparison of backbone H^α^ and H^N^ chemical shift differences between *cis* and *trans* forms is shown in Figure 6, for both solvents. Large chemical shift variation is observed for the azobenzene group and neighboring residues Arg^6^ and Thr^9^. Interestingly, more distant residues in the two peptide arms are also affected, mostly in segment 9-13 and 4-5. Similar trends are observed under both solvent conditions, albeit to a lesser extent in ACN/water. The chemical shift changes can be ascribed to magnetic susceptibility anisotropy effects as the two aromatic groups get closer in space and/or to conformational effects.

The analysis of backbone and sequential NOEs reveals the presence of complex equilibria between extended and turn/helical folded conformations, in both *cis* and *trans* forms. H^N^-H^α^ sequential NOEs are stronger than intraresidual ones, indicating that extended backbone conformations are largely populated. However, the observation of sequential H^N^-H^N^ NOEs (V4/E5, E5/R6, Y15/G16, G16/W17 in particular) can be ascribed to turn or helical conformations. These folded conformations are further supported by weak NOEs of azobenzene aromatic protons with methyl protons of Val^4^ and Leu^12^. Importantly, no NOEs could be detected between the two peptide arms in both forms. This result is in agreement with the observation that the *trans*→*cis* isomerization does not stabilize a β-hairpin structure.

## Conclusion

In this work photoisomerization of the azopeptide **1** was explored and its reversibility was compared with more simple systems, such as azobenzene amino acid **2** and azobenzene **3**. To this aim azopeptide **1** was modified introducing the photoswitch (4-aminomethyl)phenylazobenzoic acid (AMPB) to replace in [Pro7,Asn8,Thr10]CSF114 the P-N-H tripeptide on the tip of the β-hairpin.

The absorption spectra of azobenzene **3**, AMPB amino acid **2**, and azopeptide **1** were measured as a function of irradiation time, exciting into the ππ^*^ band. The major differences are observed in the case of **1**, where the isomerization results favored by exciting into the ππ^*^ transition and the corresponding cis isomer results strongly stabilized.

Detailed NMR structural studies of azopeptide **1** confirmed that the AMPB chromophore insertion into the sequence allowed reversible control of peptide conformation in solution, but the *trans*→*cis* isomerization does not stabilize a β-hairpin structure, characteristic of the original sequence. Thus, incorporation of different photocontrolled switches, such as AMPP, will require further investigations to verify their possible role in controlling β-hairpin conformations.

## Data Availability

No datasets were generated or analyzed for this study.

## Author Contributions

FN designed the experiments, performed the synthesis, interpreted the data, and wrote the manuscript. CG, RC, PRS, and GP designed, performed and interpreted the data of the UV/Vis experiments, wrote the manuscript. ML and OL designed, performed and interpreted the data of the NMR experiments, wrote the manuscript. LS provided technical support to the experiments. AMP developed the project, designed the experiments, interpreted the data, and wrote the manuscript.

### Conflict of Interest Statement

The authors declare that the research was conducted in the absence of any commercial or financial relationships that could be construed as a potential conflict of interest.
