# Supplementary material for: A Photochromic Azobenzene Peptidomimetic of a β-Turn Model Peptide Structure as a Conformational Switch
Source: Front Chem. 2019 Mar 29;7:180. doi: 10.3389/fchem.2019.00180 (PMC6449423; doi:10.3389/fchem.2019.00180)
Supplement: Supplementary file 1 [file Data_Sheet_1.docx]

**Supplementary Material**

**A photochromic azobenzene peptidomimetic of a β-turn model peptide structure as a conformational switch**

*Francesca Nuti^a,b†^, Cristina Gellini^b†^, Maud Larregola^c^, Lorenzo Squillantini^a,b^, Riccardo Chelli^b,d^, Pier Remigio Salvi^b^, Olivier Lequin^e^, Giangaetano Pietraperzia^b,d^ , Anna Maria Papini^a,b,c*^*

*^a^ Laboratory of Peptide and Protein Chemistry and Biology – PeptLab (www.peptlab.eu).*

*^b^ Department of Chemistry “Ugo Schiff”, University of Florence, Via della Lastruccia 13, 50019 Sesto Fiorentino (Italy)*

*^c^ Université Paris-Seine, PeptLab@UCP Platform and Laboratory of Chemical Biology EA4505, 5 Mail Gay Lussac, 95031 Cergy-Pontoise (France)*

*^d^* *European Laboratory for Non-Linear Spectroscopy (LENS), Via Nello Carrara 1, 50019 Sesto Fiorentino (Italy)*

*^e^ Sorbonne Université, Ecole Normale Supérieure, PSL University, CNRS, Laboratoire des Biomolécules, Paris, France*

*Corresponding author:*

*Anna Maria Papini,* [*annamaria.papini@unifi.it*](mailto:annamaria.papini@unifi.it)


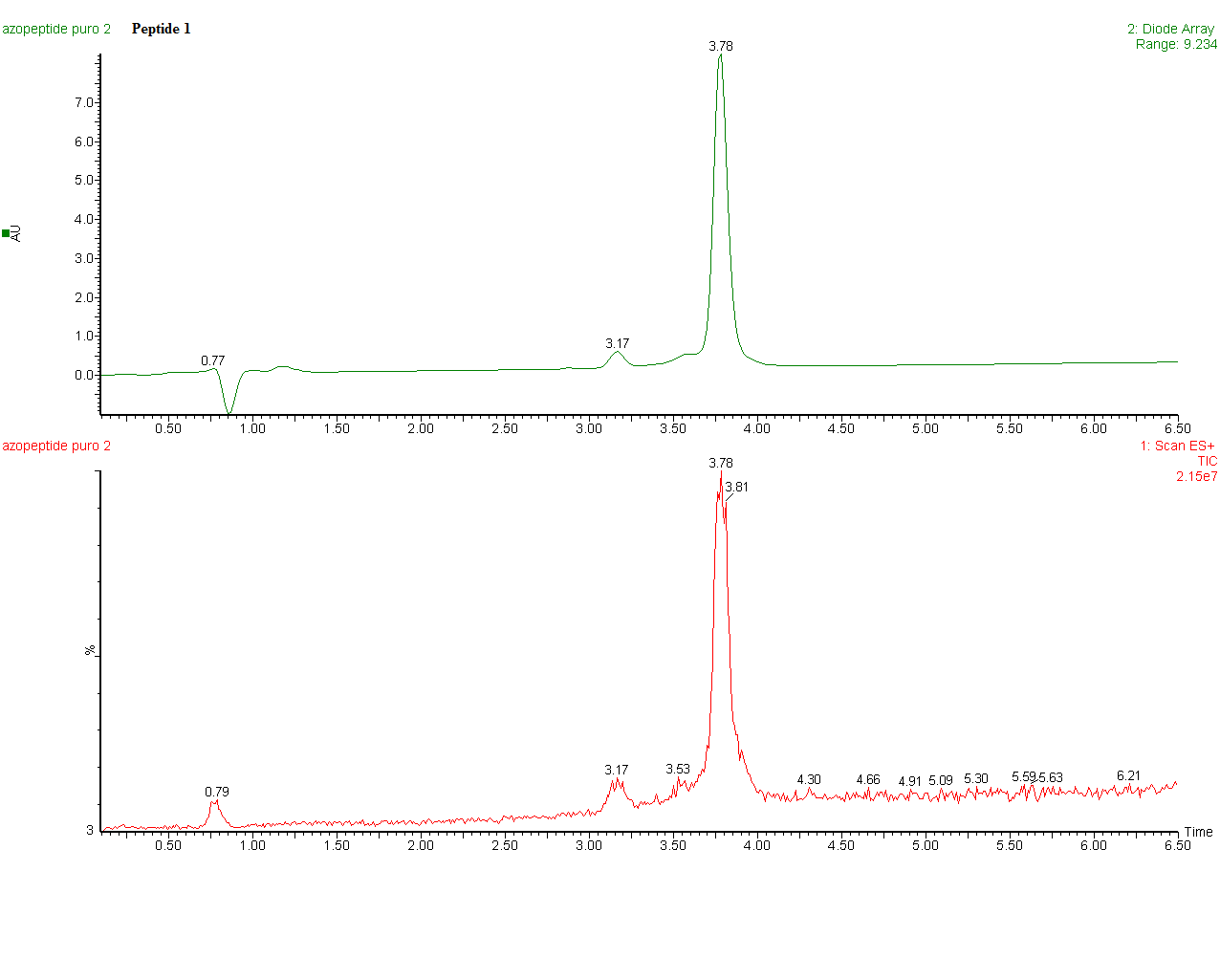


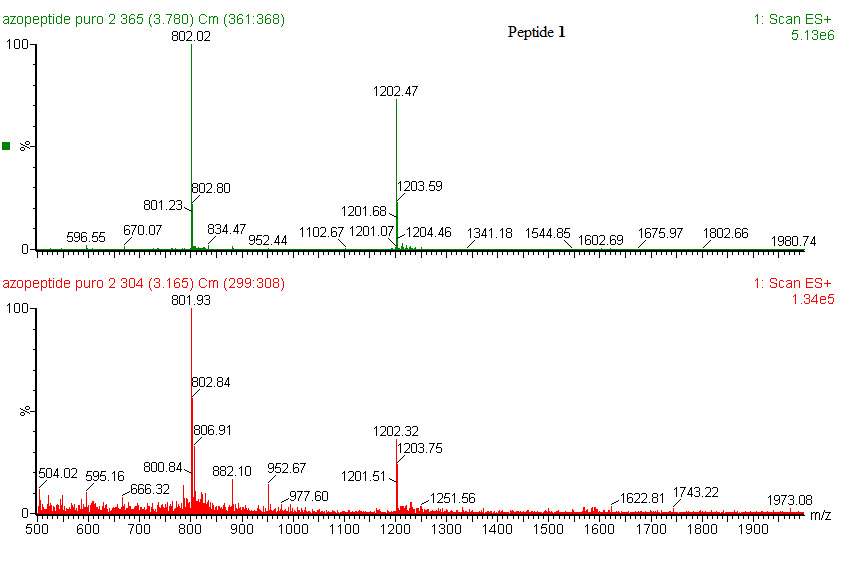


**Figure S1.** RP-HPLC: t_r_ =3.17 min (*cis* isomer) and 3.78 min (*trans* isomer), gradient 35% to 55% of B in 5 min; Mr = calcd. for C_116_ H_170_ N_29_ O_25_ S_1_: 2402,88 ESI-MS: m/z: 1202,47 [M+2H]^2+^; 802,02 [M+3H]^3+^

RP-HPLC system is an Alliance Chromatography (Waters, Milford Massachusetts, USA) with a Bioshell A160 C18 (Sigma Aldrich, Milano Italy; 1.7 μm 2.1× 50 mm) column at 35°C, at 0.6 mL/min coupled to a single quadrupole ESI-MS Micromass ZQ (Waters, Milford Massachusetts, USA).

**Figure S2.** UV-Vis absorption spectra at several irradiation time for (a) azobenzene **3**, (b) aminoazobenzene AMPB **2**. and (c) azopeptide **1**, exciting at 440 nm.

**Figure S3.** Superimposed TOCSY spectra of azopeptide **1** in TFE:H_2_O 1:1 (30°C) after 1 hour irradiation (red, 53% *cis* and 47% *trans* form) and 2 weeks relaxation (blue, 86% *trans* and 14% *cis* form). Assignments are described using one letter amino acid code. X refers to the azobenzene moiety (Azb) (see Table S1). Amino acids 1 to 20 refer to the *trans* form and 101 to 120 to the *cis* form.

**Figure S4.** NOESY spectrum (400 ms mixing time) of azopeptide **1** in TFE:D_2_O 1:1 (30°C) after 1 hour irradiation (53% *cis* and 47% *trans* form). Assignments are described using one letter amino acid code. X refers to the azobenzene moiety (Azb) (see Table S1). Amino acids 1 to 20 refer to the *trans* form and 101 to 120 to the *cis* form.

| **Table S1. ^1^H NMR assignment of the *trans* form of azopeptide 1 in TFE:H_2_O 1:1 (30 °C)**^a^ | | | | |
| --- | --- | --- | --- | --- |
| Residue | HN | H$\alpha$ | H$\beta$ | Other side chain H |
| Thr1 |  | 4.13 | 3.91 | H$\gamma2$ 1.32 |
| Pro2 | - | 4.46 | 2.26, 1.89 | H$\gamma$ 2.01, 1.96 H$\delta$ 3.71, 3.66 |
| Arg3 | 8.05 | 4.41 | 1.89, 1.78 | H$\gamma$ 1.64 H$\delta$ 3.19 |
| Val4 | 7.86 | 4.00 | 2.01 | H$\gamma$ 0.89, 0.88 |
| Glu5 | 8.57 | 4.33 | 2.05, 1.94 | H$\gamma$ 2.27 |
| Arg6 | 8.05 | 4.39 | 1.95, 1.80 |  |
| Azb7^b^ | 8.21 | 4.46 | - | H*o* 7.42 H*m* 7.78 |
| Azb8^b^ | - | 3.77 | - | H*o* 7.45 H*m* 7.80 |
| Thr9 | 7.61 | 4.30 | 4.18 | H$\gamma2$ 1.16 |
| Val10 | 7.46 | 3.94 | 1.88 | H$\gamma$ 0.72, 0.71 |
| Phe11 | 7.36 | 4.57 | 3.04, 2.87 | H$\delta$ 7.09 H$\epsilon$ 7.24 H$\zeta$ 7.18 |
| Leu12 | 7.38 | 4.34 | 1.48, 1.40 | H$\delta$ 0.79 |
| Ala13 | 7.60 | 4.38 | 1.24 |  |
| Pro14 | - | 4.19 | 2.07, 1.57 | H$\gamma$ 1.79 H$\delta$ 3.57, 3.33 |
| Tyr15 | 7.22 | 4.34 | 2.92, 2.79 | H$\delta$ 6.99 H$\epsilon$ 6.82 |
| Gly16 | 8.02 | 3.93, 3.74 |  |  |
| Trp17 | 7.49 | 4.62 | 3.33, 3.23 | H$\delta1$ 7.20 H$\epsilon1$ 9.70 H$\epsilon3$ 7.51 H$\zeta2$ 7.39 H$\zeta3$ 7.09 H$\eta2$ 7.17 |
| Met18 | 7.70 | 4.31 | 1.92, 1.81 | H$\gamma$ 2.12 |
| Val19 | 7.47 | 4.10 | 2.08 | H$\gamma$ 0.89 |
| Lys20 | 7.56 | 4.23 | 1.84, 1.74 | H$\gamma$ 1.39 H$\delta$ 1.68 H$\epsilon$ 2.97 |
| ^a^ ^1^H chemical shifts are referenced to the residual protiated solvent signal (δ ^1^H 3.88 ppm).  ^b^ Azb7 and Azb8 are defined as moieties linked to N=N group: H$\alpha$ protons correspond to CH_2_ groups; aromatic *ortho* (H*o*) and *meta* (H*m*) positions are named with respect to the CH_2_ substituent. | | | | |

| **Table S2. ^1^H NMR assignment of the *cis* form of azopeptide 1 in TFE:H_2_O 1:1 (30 °C).**^a^ Only residues showing ^1^H chemical shift differences with the *trans* form are reported. | | | | |
| --- | --- | --- | --- | --- |
| Residue | HN | H$\alpha$ | H$\beta$ | Other side chain H |
| Val4 | 7.88 | 4.07 | 2.02 | H$\gamma$ 0.90, 0.89 |
| Glu5 | 8.47 | 4.33 | 2.02, 1.90 | H$\gamma$ 2.23 |
| Arg6 | 8.04 | 4.34 | 1.89, 1.76 | H$\gamma$ 1.62 H$\delta$ 3.15 |
| Azb7^b^ | 8.12 | 4.30, 4.27 | - | H*o* 7.15 H*m* 6.82 |
| Azb8^b^ | - | 3.63 | - | H*o* 7.21 H*m* 6.83 |
| Thr9 | 7.67 | 4.22 | 4.15 | H$\gamma2$ 1.11 |
| Val10 | 7.43 | 4.01 | 1.90 | H$\gamma$ 0.70, 0.69 |
| Phe11 | 7.57 | 4.63 | 3.11, 2.87 | H$\delta$ 7.10 H$\epsilon$ 7.24 H$\zeta$ 7.17 |
| Leu12 | 7.51 | 4.37 | 1.52 | H$\delta$ 0.80 |
| Ala13 | 7.67 | 4.43 | 1.24 |  |
| Pro14 | - | 4.21 | 2.10, 1.61 | H$\gamma$ 1.85 H$\delta$ 3.61, 3.39 |
| Tyr15 | 7.20 | 4.35 | 2.92, 2.81 | H$\delta$ 6.99 H$\epsilon$ 6.82 |
| Trp17 | 7.49 | 4.65 | 3.33, 3.23 |  |
| Met18 | 7.74 | 4.33 | 1.92, 1.81 | H$\gamma$ 2.15 |
| Val19 | 7.50 | 4.12 | 2.08 | H$\gamma$ 0.91 |
| ^a^ ^1^H chemical shifts are referenced to the residual protiated solvent signal  (δ ^1^H 3.88 ppm)  ^b^ Azb^7^ and Azb^8^ are defined as moieties linked to N=N group: H$\alpha$ protons correspond to CH_2_ groups; aromatic *ortho* (H*o*) and *meta* (H*m*) positions are named with respect to the CH_2_ substituent. | | | | |

| **Table S3. ^1^H NMR assignment of the *trans* form of azopeptide 1 in ACN:H_2_O 1:1 (25 °C)**^a^ | | | | |
| --- | --- | --- | --- | --- |
| Residue | HN | H$\alpha$ | H$\beta$ | Other side chain H |
| Thr1 |  | 3.93 | 3.75 | H$\gamma2$ 1.17 |
| Pro2 | - | 4.33 | 2.16, 1.77 | H$\gamma$ 1.88, 1.85 H$\delta$ 3.64, 3.56 |
| Arg3 | 8.01 | 4.24 | 1.74, 1.61 | H$\gamma$ 1.49 H$\delta$ 3.06 |
| Val4 | 7.86 | 3.92 | 1.90 | H$\gamma$ 0.78, 0.77 |
| Glu5 | 8.41 | 4.18 | 1.93, 1.79 | H$\gamma$ 2.13 |
| Arg6 | 7.98 | 4.25 | 1.81, 1.66 | H$\gamma$ 1.50 H$\delta$ 3.07 |
| Azb7^b^ | 8.21 | 4.37 | - | H*o* 7.39 H*m* 7.74 |
| Azb8^b^ | - | 3.71 | - | H*o* 7.45 H*m* 7.76 |
| Thr9 | 7.84 | 4.19 | 4.10 | H$\gamma2$ 1.05 |
| Val10 | 7.52 | 3.95 | 1.81 | H$\gamma$ 0.61, 0.61 |
| Phe11 | 7.61 | 4.47 | 3.00, 2.73 | H$\delta$ 7.07 H$\epsilon$ 7.17 H$\zeta$ 7.14 |
| Leu12 | 7.52 | 4.19 | 1.40 | H$\delta$ 0.70, 0.69 |
| Ala13 | 7.64 | 4.36 | 1.13 |  |
| Pro14 | - | 4.13 | 1.98, 1.50 | H$\gamma$ 1.74, 1.72 H$\delta$ 3.50, 3.35 |
| Tyr15 | 7.39 | 4.29 | 2.89, 2.72 | H$\delta$ 6.95 H$\epsilon$ 6.69 |
| Gly16 | 7.82 | 3.82, 3.60 |  |  |
| Trp17 | 7.47 | 4.50 | 3.13 | H$\delta1$ 7.10 H$\epsilon1$ 9.90 H$\epsilon3$ 7.44 H$\zeta2$ 7.31 H$\zeta3$ 6.98 H$\eta2$ 7.05 |
| Met18 | 7.67 | 4.18 | 1.84, 1.70 | H$\gamma$ 2.11 H$\epsilon$ 1.92 |
| Val19 | 7.50 | 4.04 | 1.96 | H$\gamma$ 0.79, 0.78 |
| Lys20 | 7.53 | 4.06 | 1.68, 1.58 | H$\gamma$ 1.25 H$\delta$ 1.52 H$\epsilon$ 2.83 |
| ^a^ ^1^H chemical shifts are referenced to the residual protiated solvent signal (δ ^1^H 1.94 ppm)  ^b^ Azb^7^ and Azb^8^ are defined as moieties linked to N=N group: H$\alpha$ protons correspond to CH_2_ groups; aromatic *ortho* (H*o*) and *meta* (H*m*) positions are named with respect to the CH_2_ substituent. | | | | |

| **Table S4. ^1^H NMR assignment of the *cis* form of azopeptide 1 in ACN:H_2_O 1:1 (25 °C).**^a^ Only residues showing ^1^H chemical shift differences with the *trans* form are reported. | | | | |
| --- | --- | --- | --- | --- |
| Residue | HN | H$\alpha$ | H$\beta$ | Other side chain H |
| Val4 | 7.87 | 3.95 | 1.90 | H$\gamma$ 0.79, 0.77 |
| Glu5 | 8.35 | 4.18 | 1.90, 1.75 | H$\gamma$ 2.09 |
| Arg6 | 7.97 | 4.19 | 1.74, 1.60 | H$\gamma$ 1.47 H$\delta$ 3.04 |
| Azb7^b^ | 8.13 | 4.21 | - | H*o* 7.10 H*m* 6.76 |
| Azb8^b^ | - | 3.55 | - | H*o* 7.17 H*m* 6.77 |
| Thr9 | 7.81 | 4.16 | 4.05 | H$\gamma2$ 0.98 |
| Val10 | 7.54 | 3.98 | 1.84 | H$\gamma$ 0.63, 0.61 |
| Phe11 | 7.72 | 4.50 | 3.01, 2.75 | H$\delta$ 7.07 |
| Leu12 | 7.58 | 4.20 | 1.41 | H$\delta$ 0.69 |
| Ala13 | 7.68 | 4.37 | 1.14 |  |
| ^a^ ^1^H chemical shifts are referenced to the residual protiated solvent signal (δ ^1^H 1.94 ppm)  ^b^ Azb^7^ and Azb^8^ are defined as moieties linked to N=N group: H$\alpha$ protons correspond to CH_2_ groups; aromatic *ortho* (H*o*) and *meta* (H*m*) positions are named with respect to the CH_2_ substituent. | | | | |
